# Nerve Root Compression Due to Contralateral Compensatory Hypertrophy: A Rare Case of Vertebral Artery Thrombosis

**DOI:** 10.5334/jbsr.3169

**Published:** 2023-09-04

**Authors:** Ertugrul Cakir, Merve Nur Tasdemir, Alptekin Tosun

**Affiliations:** 1 Giresun University, Faculty of Medicine, Department of Radiology, Giresun, TR

**Keywords:** Thrombosis, Vertebral Artery, Nerve Root, Compression, Compensatory, Computed Tomography Angiography

## Abstract

In the patient who complained of dizziness, thrombus was observed in the right vertebral artery. Compression was detected in the nerve root due to compensatory hypertrophy in the left vertebral artery.

In conclusion, in cases of unilateral vertebral artery thrombosis, should be aware of the pathologies that may develop in adjacent structures due to compensatory hypertrophy.

**Teaching Point:**

An occluded vertebral artery (VA) can cause contralateral VA hypertrophy, which can cause various pathologies including nerve root compression.

## Case History

**Figure 1:**
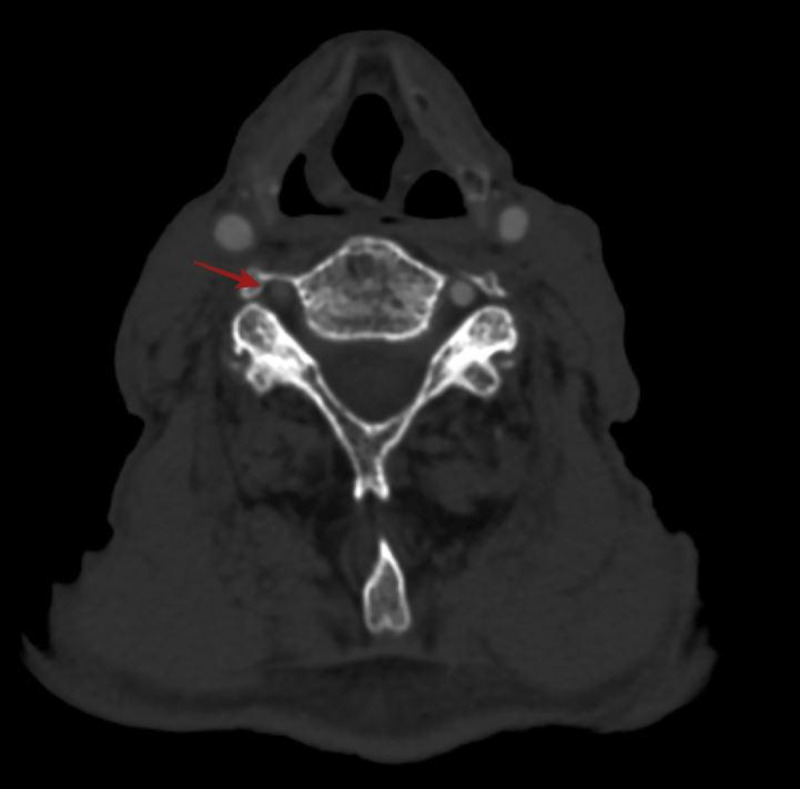


**Figure 2:**
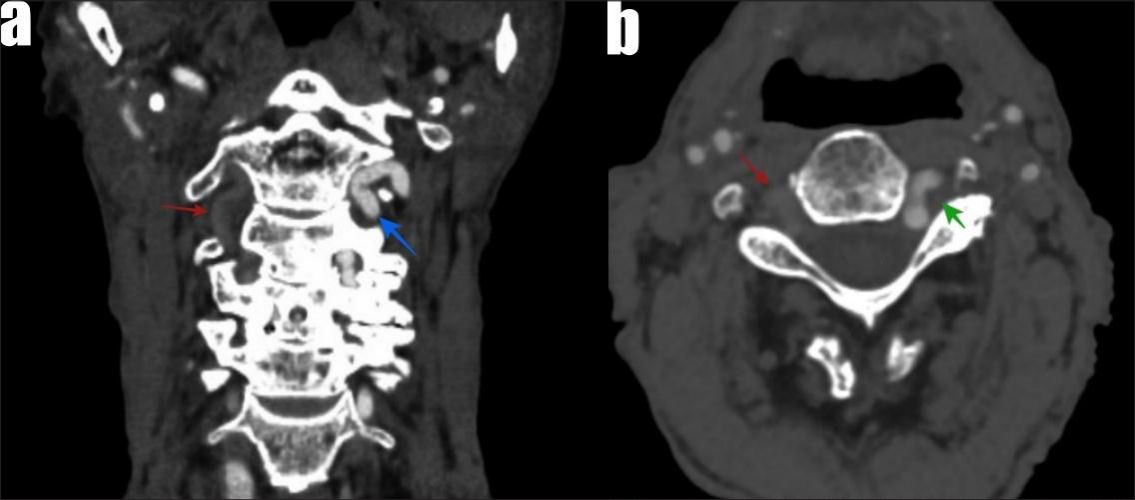


A 79-year-old male patient was admitted to the hospital with complaints of dizziness. Computed tomography angiography showed no contrast opacification of the right vertebral artery (VA) (red arrow in Figure 1), while there was hypertrophy of the left VA. Thrombosis of the right VA (red arrow) and a compensatory hypertrophic left VA loop (blue arrow) compressing the nerve root (green arrow) in the neural foraminal area were observed (Figure 2). Retrospective patient’s history revealed that he had numbness of the left arm. Nerve root compression was detected incidentally by this radiological examination.

VA loop is a rare vascular condition that may be associated with nerve root compression and may occur due to congenital or acquired causes. Usually, symptoms such pain, numbness, and decreased muscle strength result from cervical discopathies and spondylosis-associated cervical nerve compression. However, compensatory vascular hypertrophy secondary to controlateral thrombosis as in our case is very rarely reported in the literature [1]. The therapeutic management may be adjusted following the diagnosis.

In conclusion, in cases of unilateral VA thrombosis, secondary pathology may develop in adjacent structures due to chronic compression by a benign hypertrophic vascular structure.
